# Myocardial Contractility Pattern Characterization in Radiation-Induced Cardiotoxicity Using Magnetic Resonance Imaging: A Pilot Study with ContractiX

**DOI:** 10.3390/tomography9010004

**Published:** 2022-12-22

**Authors:** El-Sayed H. Ibrahim, Antonio Sosa, Sherry-Ann Brown, Dayeong An, Slade Klawikowski, John Baker, Carmen Bergom

**Affiliations:** 1 Department of Radiology, Medical College of Wisconsin, 8701 Watertown Plank Rd, Milwaukee, WI 53226, USA; 2 Department of Medicine, Medical College of Wisconsin, 8701 Watertown Plank Rd, Milwaukee, WI 53226, USA; 3 Department of Biomedical Engineering, Marquette University, 1250 W Wisconsin Ave, Milwaukee, WI 53233, USA; 4 Department of Radiation Oncology, Medical College of Wisconsin, 8701 Watertown Plank Rd, Milwaukee, WI 53226, USA; 5 Department of Surgery, Medical College of Wisconsin, 8701 Watertown Plank Rd, Milwaukee, WI 53226, USA; 6 Department of Radiation Oncology, Washington University, 1 Brookings Dr, St. Louis, MO 63130, USA

**Keywords:** MRI, thoracic cancer, cardiac function, radiation therapy, strain imaging, cardiotoxicity, ContractiX

## Abstract

Radiation therapy (RT) plays an integral role in treating thoracic cancers, despite the risk of radiation-induced cardiotoxicity. We hypothesize that our newly developed magnetic resonance imaging (MRI)-based contractility index (ContractiX) is a sensitive marker for early detection of RT-induced cardiotoxicity in a preclinical rat model of thoracic cancer RT. Adult salt-sensitive rats received image-guided heart RT and were imaged with MRI at 8 weeks and 10 weeks post-RT or sham. The MRI exam included cine and tagging sequences to measure left-ventricular ejection fraction (LVEF), mass, myocardial strain, and ContractiX. Furthermore, ventricular torsion, diastolic strain rate, and mechanical dyssynchrony were measured. Statistical analyses were performed between the sham, 8 weeks post-RT, and 10 weeks post-RT MRI parameters. The results showed that both LVEF and myocardial mass increased post-RT. Peak systolic strain and ContractiX significantly decreased post-RT, with a more relative reduction in ContractiX compared to strain. ContractiX showed an inverse nonlinear relationship with LVEF and continuously decreased with time post-RT. While early diastolic strain rate and mechanical dyssynchrony significantly changed post-RT, ventricular torsion changes were not significant post-RT. In conclusion, ContractiX measured via non-contrast MRI is a sensitive early marker for the detection of subclinical cardiac dysfunction post-RT, and it is superior to other MRI cardiac measures.

## 1. Introduction

Along with systemic therapies, radiation therapy (RT) plays an integral role in treating advanced lung cancer. However, the incidence of RT-induced cardiac complications in this patient population is as high as 33% [1,2,3]. Recent studies suggest that radiation-induced cardiac damage can manifest within two years of completing RT for lung cancer [1,4,5,6,7,8,9] and that mortality correlates with mean heart dose [4] or with the percentage of the heart receiving 5 Gy [10], 30 Gy and/or 50 Gy [8]. A national multicenter lung cancer trial and other single-institution studies have found correlations between early death and radiation dose to the heart [11]. Interestingly, many trials have not been able to correlate early deaths with clinically evident cardiotoxicity, suggesting that early non-cancer deaths may be partially due to subclinical cardiac dysfunction.

The current paradigm for cardiac function assessment and management of cardiovascular disease relies primarily upon the assessment of global cardiac function, e.g., left ventricle ejection fraction (LVEF) [12,13]. However, LVEF alone is limited in its diagnostic and prognostic ability, as ventricular remodeling can compensate for regional cardiac dysfunction to maintain global function and cardiac output in acute and subacute phases post-RT before LVEF ultimately declines [14,15,16]. Thus, LVEF may be stable or even increases after radiation exposure due to compensatory mechanisms [16]. Therefore, identifying new markers capable of early detection of subclinical cardiac dysfunction after cardiac RT exposure is essential for risk stratification and prompt initiation of cardioprotective heart therapy to avoid non-reversible late cardiac damage [17].

Magnetic resonance imaging (MRI) tagging [18] allows for quantitative assessment of myocardial contractility on a regional basis. As regional changes in myocardial contractility frequently occur before cardiac damage [14,15], regional cardiac dysfunction is potentially reversible, and myocardial strain-derived parameters can be early indicators of cardiotoxicity. This has been demonstrated in studies where MRI-generated strain parameters detected abnormal myocardial function in cancer patients treated with chemotherapy, despite normal systolic function [19,20,21,22,23,24,25]. Recent results from our preclinical work identified heterogeneous contractility patterns in different LV regions with reduced myocardial strain post-RT [15,16].

In this study, we investigated the capability of advanced MRI-generated parameters of heart mechanics for characterizing the myocardial contractility patterns post-RT and for early detection of RT-induced cardiotoxicity in a pre-clinical rat model of radiation-induced cardiotoxicity. We present the contractility index (ContractiX) derived from regional strain parameters. We hypothesize that ContractiX is a sensitive marker for early detection of RT-induced cardiotoxicity. We also examined the effect of RT on other MRI-generated heart contractility parameters, including ventricular torsion, diastolic strain rate, and mechanical dyssynchrony.

## 2. Materials and Methods

### 2.1. Animal Model of Radiation Therapy

This study was approved by the Medical College of Wisconsin Institutional Animal and Use Care Committee. Adult female Dahl salt-sensitive (SS) rats, aged ~10 weeks, were randomized into two groups: sham-treated (N = 7), that were imaged at 8 weeks post-sham, and RT-treated (N = 5) that were imaged at both 8 weeks and 10 weeks post-RT, as previously described [15]. The RT group received image-guided localized whole-heart RT to 24 Gy using three equally-weighted fields with a 1.5 cm collimator, as previously described (1 anterior-posterior beam and two lateral beams, 225 kVp, 13 mA, 0.32 mm Cu, 2.69 Gy/min) [16,26]. Briefly, the animals were anesthetized using 3% isoflurane and inhaled room-temperature air. An image-guided X-RAD SmART irradiator (Precision X-Ray, Madison, CT, USA), which was regularly verified for output using a calibrated ionization chamber, was used for the RT. Doses were calculated using a Monte-Carlo-based system (MAASTRO Radiotherapy Clinic, Maastricht, Netherlands) [16,26].

### 2.2. MRI Scans

The rats were imaged on a small-animal 9.4T MRI scanner with a 30-cm bore diameter (Bruker, Rheinstetten, Germany) using a 4-element surface coil [27]. The MRI scan included both long-axis (LAX) and short-axis (SAX) cine and tagged images acquired using fast low-angle shot (FLASH) pulse sequences with both cardiac and respiratory gating. A stack of SAX cine slices covering the whole LV and three SAX-tagged slices (basal, mid-ventricular, and apical) were acquired. The cine sequence imaging parameters were the following: repetition time (TR) = 7 ms, echo time (TE) = 2.1 ms, flip angle = 15°, matrix = 176 *×* 176, the field of view (FOV) = 40 mm × 40 mm, slice thickness = 1 mm, acquisition bandwidth = 526 Hz/pixel, #averages = 2, #cardiac phases = 20, scan time ~2 min/slice. The tagging sequence imaging parameters were similar to cine imaging, except for the following: matrix = 256 × 256, acquisition bandwidth = 375 Hz/pixel, #averages = 3, scan time = 4–5 min/slice.

### 2.3. Image Analysis and Statistics

The cine images were analyzed using the cvi42 (Circle Cardiovascular Imaging, Calgary, Canada) software to measure left ventricle (LV) end-diastolic volume (EDV), end-systolic volume (ESV), stroke volume (SV), EF, and mass. The tagged images were analyzed using the SinMod [28] technique (InTag, Lyon, France) to measure myocardial circumferential, radial, and longitudinal peak-systolic strains (Ecc, Err, Ell) in different LV segments based on the American Heart Association (AHA) 17-segment model. The reproducibility of the SinMod technique has been previously shown by our group [29]. Strain analysis in this work was conducted three times: twice by the same observer with >2 months between the two analyses to assess intra-observer variability; and once by another observer to assess inter-observer variability. For clarity of presentation, strain measurements are presented in absolute value (i.e., circumferential and longitudinal strain measurements, which are originally negative due to tissue contraction in these directions during systole, are represented by their absolute values).

### 2.4. The ContractiX Parameter

The ContractiX parameter is introduced as a new measure of heart contractility performance derived from regional strain measurements in different segments in the LV AHA segmental model. First, the strain value for each rat was calculated as the mean of peak systolic strain from all segments. ContractiX was generated as the percentage of normally contracting myocardium for each rat, which was calculated as the number of normally contracting segments, divided by the total number of segments, and multiplied by 100; therefore, ContractiX ranges from 0 (none of the myocardial segments is normally contracting) to 100 (all myocardial segments are normally contracting). A myocardial segment was considered normally contracting if its peak systolic strain exceeded a threshold value, calculated as the mean minus one standard deviation (SD) of the strain values from all sham rats. ContractiX values were calculated from different strain components (circumferential (ContractiX-circ), longitudinal (ContractiX-long), and radial (ContractiX-rad)) and changes in ContractiX post-RT were compared to those in LVEF and strain values. ContractiX was also calculated from strains in more than one direction, starting with circumferential and longitudinal directions (ContractiX-CL) and then all three directions (ContractiX-CLR).

### 2.5. Other Contractility Parameters

Ventricular torsion, another parameter of systolic function, was measured as the difference between basal and apical rotation angles at peak systole divided by the distance between the basal and apical slices. Besides systolic function, early-diastolic strain rate (SR) was calculated in different directions (SR-cc (circumferential), SR-rr (radial), SR-ll (longitudinal)). For presentation clarity, diastolic SR measures are represented in absolute values. Finally, to assess myocardial mechanical dyssynchrony, the standard deviations of time-to-peak (TTP) systolic strain between different myocardial segments, based on the AHA 17-segment model, were calculated in different directions (TTP-cc, TTP-rr, TTP-ll).

### 2.6. Statistical Analysis

All measurements were represented as mean ± standard deviation (SD). ANOVA analysis with the Tukey post hoc test was conducted for multiple comparisons between different rat groups. Spearman correlation analysis was conducted between ContractiX and LVEF. *p* < 0.05 was considered significant. Bland-Altman analysis [30] was conducted to assess intra-observer and inter-observer variabilities in the generated measurements.

## 3. Results

### 3.1. LVEF and Mass Are Increased Post-RT

As shown in Table 1, LVEF significantly increased post-RT compared to the sham rats, as previously reported [15]. The decrease in EDV post-RT was accompanied by a corresponding decrease in ESV post-RT, which resulted in maintained SV at all time points. Furthermore, LV mass significantly increased post-RT compared to the sham rats.

### 3.2. Strain Is Reduced Post-RT

**Figure 1:**
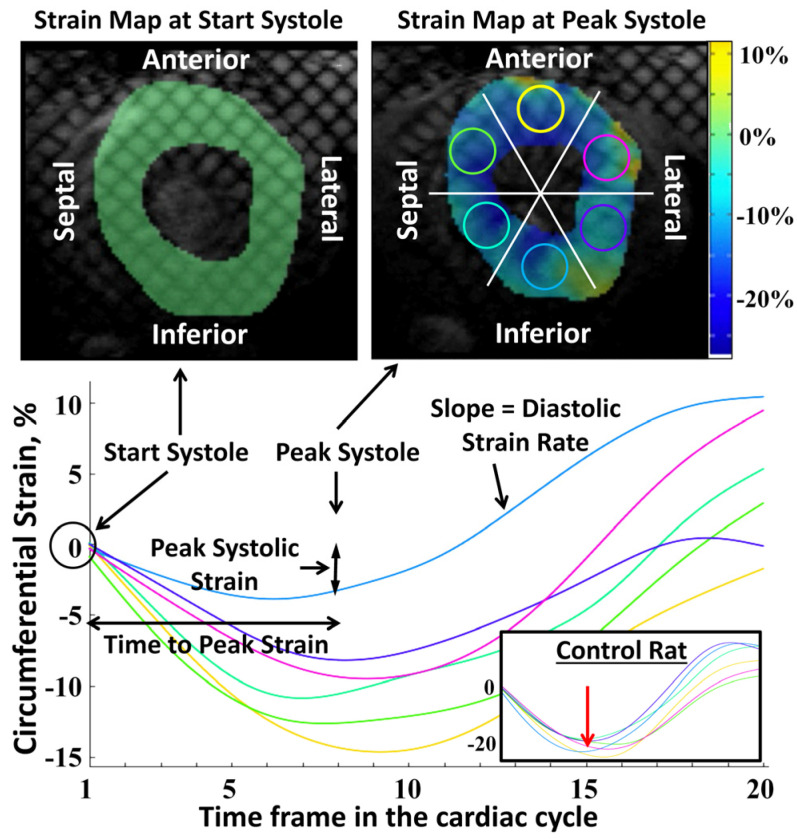
Radiation causes decreased and heterogeneous regional myocardial strain. Tagged images and strain curves of a rat model of RT, showing radiation effect on regional strain distribution at 8 weeks post-RT. The curves show strain values throughout the cardiac cycle (20 timeframes) from six segments (**top right** image) in a mid-ventricular short-axis slice. The color of each curve matches the color of the corresponding circular region in the myocardium (**top right**). At start-systole (**top left**), strain is zero at all segments (tagging grid is not deformed). At peak-systole (**top right**), the tagging grid is not uniformly deformed, where the myocardium color represents regional strain value based on the strain color bar. Note that the strain curves show different peak strains in different segments (colored circles in the **top right**). The figure also shows ‘peak systolic strain’, ‘time-to-peak strain’ and ‘diastolic strain rate’ parameters for the inferior segment (blue curve). The inset in the **bottom right** shows an example of strain curves from a sham control rat where all strain curves (different myocardial regions) come to a large peak strain value at the same time point (red arrow).

**Figure 2:**
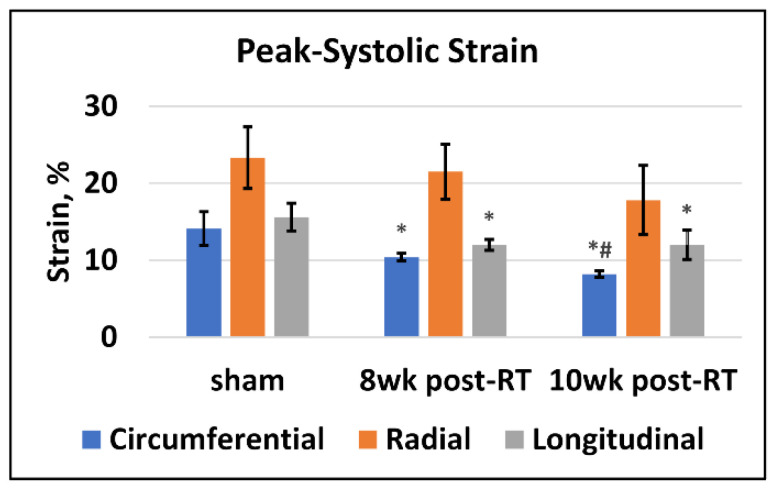
Peak systolic strain is reduced post-RT. Values are represented as mean ± standard deviation (black error bars). Peak systolic strain in the circumferential (blue), radial (orange), and longitudinal (gray) directions in the sham and RT rats at 8 weeks and 10 weeks post-RT. For display purposes, circumferential and longitudinal strains are represented in absolute values (original values are negative). Asterisk (*) and hash (#) symbols represent statistically significant (*p* < 0.05) measurement differences with respect to sham and 8-weeks post-RT measurements, respectively. Circumferential and longitudinal strains show significant reductions post-RT compared to sham. Furthermore, circumferential measurements show a significant reduction at 10 weeks post-RT compared to 8 weeks post-RT.

**Figure 3:**
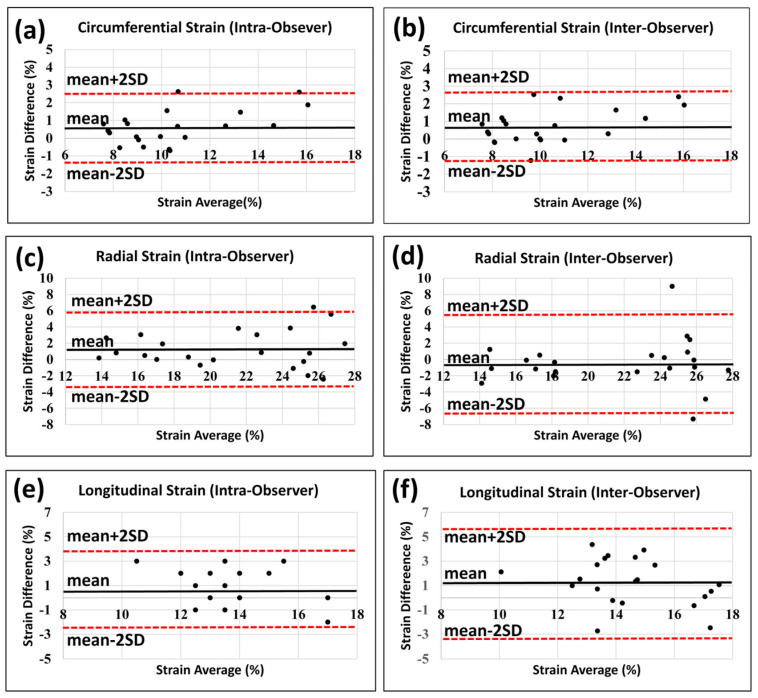
Bland-Altman plots show low intra- and inter-observer variabilities in strain measurements. Strain analysis was conducted twice by the same observer with >2 months between analyses to assess intra-observer variability and once by another observer to assess inter-observer variability. Intra-observer (**a**,**c**,**e**) and inter-observer (**b**,**d**,**f**) measurements for circumferential (**a**,**b**), radial (**c**,**d**), and longitudinal (**e**,**f**) strains show low variabilities. All measurement differences lay within the mean ± 2 SD range, except for one point in (**c**) and one point in (**d**) in the radial intra-observer and inter-observer measurements, respectively.

Figure 1 shows representative tagged images and generated strain curves in an RT rat at 8 weeks post-RT. Despite increased LVEF, different strain measurements (Ecc, Err, Ell) decreased post-RT, as shown in Table 1 and Figure 2. Bland-Altman analysis (Figure 3) demonstrated low intra- and inter-observer variabilities of strain measurements in different directions, where almost all measurement differences lay within the agreement range of mean ± 2 SD of the measurement differences.

### 3.3. ContractiX Is a Sensitive Early Marker of Subclinical Cardiac Dysfunction Post-RT

**Figure 4:**
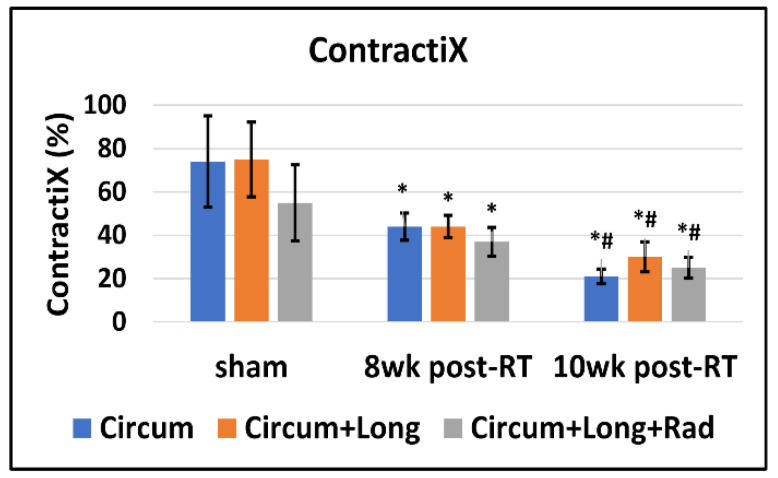
ContractiX is significantly decreased post-RT. Values are represented as mean ± standard deviation (black error bars). ContractiX (representing % normally contracting myocardium) calculated from circumferential (blue), circumferential plus longitudinal (orange), and circumferential plus longitudinal plus radial (gray) strain measurements in sham and RT rats at 8 weeks and 10 weeks post-RT. Asterisk (*) and hash (#) symbols represent statistically significant (*p* < 0.05) measurement differences with respect to sham and 8 weeks post-RT measurements, respectively. All ContractiX parameters show significant reductions post-RT compared to sham, as well as significant reductions at 10 weeks post-RT compared to 8 weeks post-RT.

**Figure 5:**
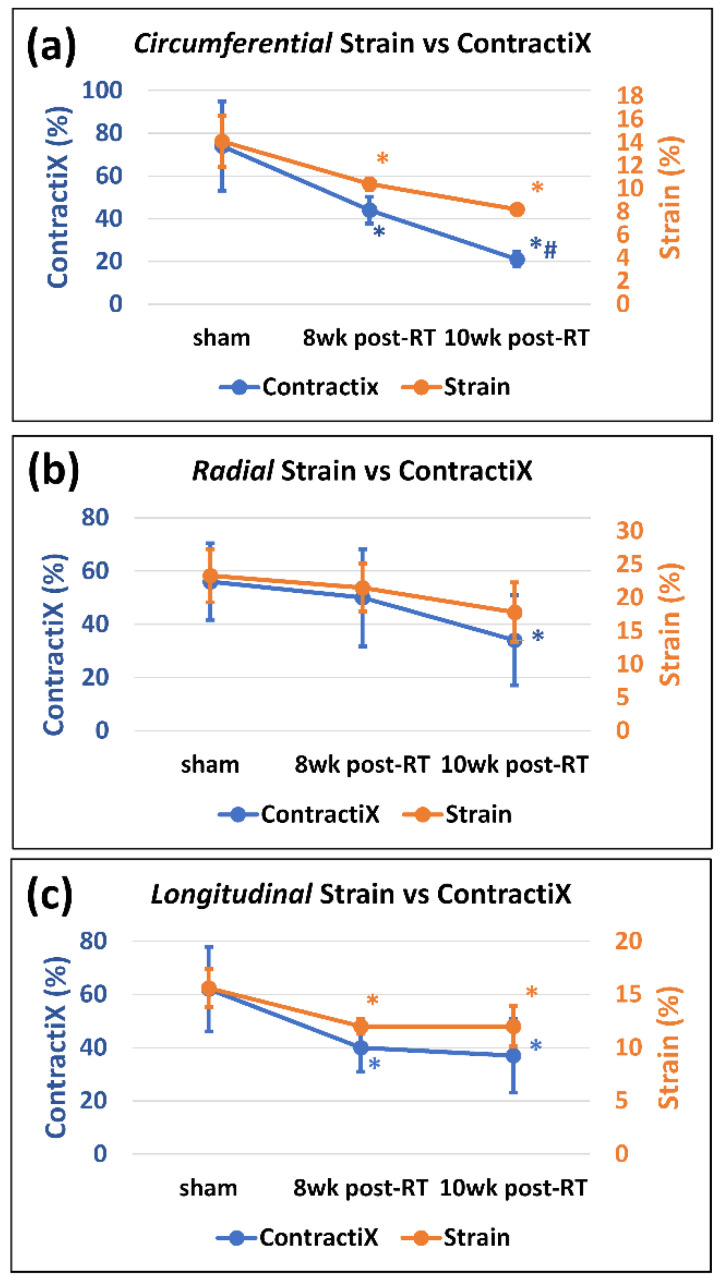
ContractiX shows larger reductions post-RT compared to strain reductions. Values are represented as mean ± standard deviation (black error bars).Peak systolic strain (orange) and ContractiX (blue) measurements in the (**a**) circumferential, (**b**) radial, and (**c**) longitudinal directions in sham and RT rats at 8 weeks and 10 weeks post-RT. For display purposes, circumferential and longitudinal strains are represented in absolute values (original values are negative). Note more reduction in ContractiX than in strain post-RT. Asterisk (*) and hash (#) symbols represent statistically significant (*p* < 0.05) measurement differences with respect to sham and 8 weeks post-RT measurements, respectively.

The ContractiX parameter significantly reduced post-RT in all directions, as shown in Table 1, Figure 4 and Figure 5, where the degree of reduction was most in the circumferential direction, followed by that in the longitudinal direction, and then in the radial direction. Nevertheless, the degree of reduction in ContractiX was always larger than that in strain in all directions, as shown in Figure 5. Note the capability of ContractiX not only for demonstrating significant differences between measurements at 8 weeks and 10 weeks post-RT compared to sham but also for significantly differentiating between measurements at 10 weeks post-RT compared to 8 weeks post-RT (Figure 4), showing its high sensitivity for detecting progression in cardiac dysfunction post-RT.

**Figure 6:**
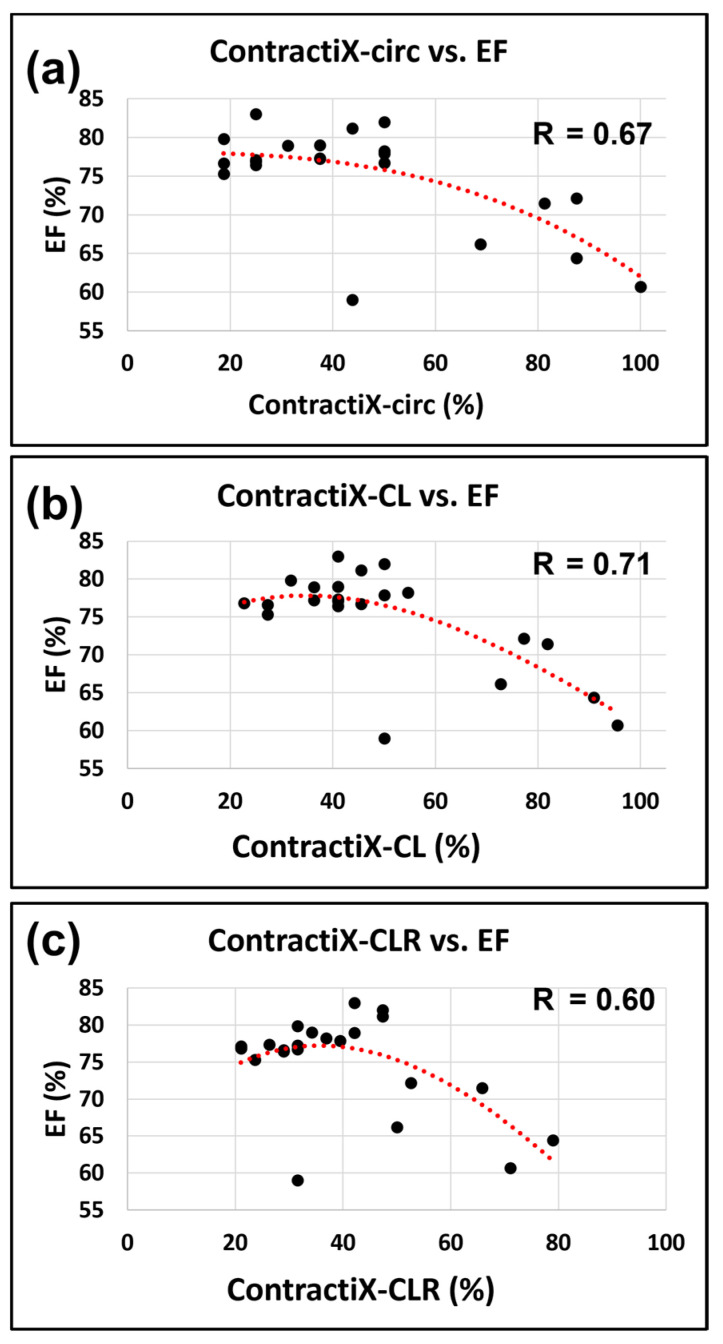
ContractiX shows a nonlinear relationship with LVEF. Scatter plots of ContractiX calculated from (**a**) circumferential (ContractiX-circ), (**b**) circumferential plus longitudinal (ContractiX-CL), and (**c**) circumferential plus longitudinal plus radial (ContractiX-CLR) strains versus ejection fraction (EF) in all scanned rats. While EF is ≥59% in all rats, ContractiX spans a wide range of measurements between 25% and 100%, and it shows a nonlinear inverse relationship with EF. The correlation coefficients (R) are shown in the figure, and the *p*-value was <0.05 for all cases (note the overlap of some points from different animals).

While normal LVEF was maintained post-RT, all ContractiX parameters showed a continuous decrease post-RT. This can be explained by the nonlinear inverse relationship between LVEF and ContractiX (Figure 6), in which ContractiX spans a wide range of measurements between 25% and 100% in rats in different groups despite normal LVEF (≥59%) in all rats. Correlation analysis showed a good fit between ContractiX and LVEF, as shown by the large R values in Figure 6. Compared to the ContractIX values shown in Table 1 (ContractiX-circ, ContractiX-rad, ContractiX-long), when calculating ContractiX using both circumferential and longitudinal strains, ContractiX-CL decreased from 75 ± 17.2% in the sham rats to 44 ± 5.2% (*p* < 0.01) and 30 ± 6.9% (*p* < 0.01) at 8-weeks and 10-weeks post-RT, respectively, where the degree of reduction post-RT lied between those of ContractiX-circ and ContractiX-long. When calculating ContractiX using all strain components (circumferential, longitudinal, and radial), ContractiX-CLR decreased from 55 ± 17.6% in the sham rats to 37 ± 6.7% (*p* = 0.03) and 25 ± 4.8% (*p* < 0.01) at 8-weeks and 10-weeks post-RT, respectively.

### 3.4. Other Heart Contractility Parameters Are Affected Post-RT

**Figure 7:**
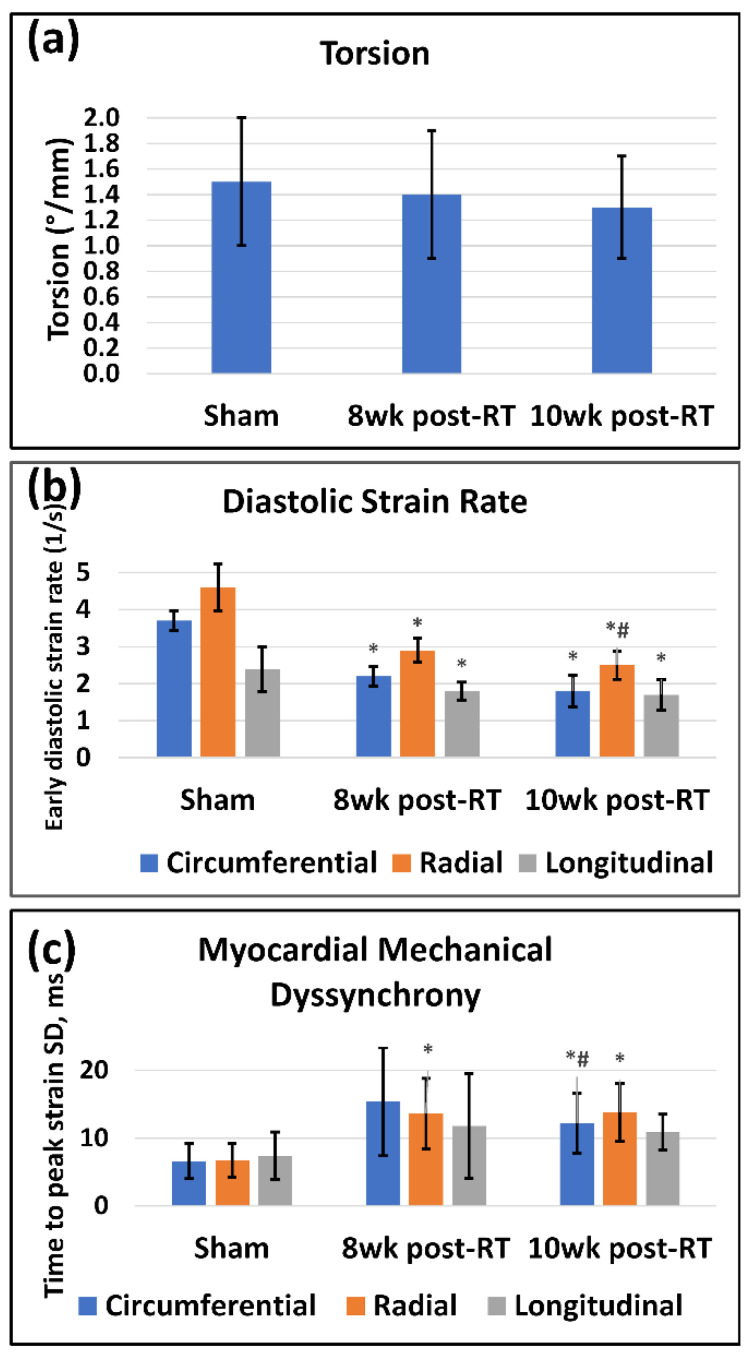
MRI-derived contractility parameters are affected by RT. Values are represented as mean ± standard deviation (black error bars). (**a**) Torsion (°/mm) is measured as the difference between basal and apical rotation angles, divided by the distance between the two slices. Ventricular torsion showed a non-significant decrease at 8 weeks post-RT (compared to sham values), then increased at 10 weeks post-RT (but to a lower value than that in sham rats). (**b**) Early diastolic strain rate (1/s) in the circumferential (blue), radial (orange), and longitudinal (gray) directions in sham, 8 weeks post-RT, and 10 weeks post-RT rats. For presentation purposes, radial diastolic strain rates are shown in absolute values (original values are negative). (**c**) Myocardial mechanical dyssynchrony, represented by the standard deviation of time to peak systolic circumferential (blue), radial (orange), and longitudinal (gray) strains between different myocardial segments (based on the AHA 17-segment model in sham, 8 weeks post-RT, and 10 weeks post-RT rats). Asterisk (*) and hash (#) symbols represent statistically significant (*p* < 0.05) measurement differences with respect to sham and 8 weeks post-RT measurements, respectively.

Besides strain, other heart contractility parameters also demonstrated worsening cardiac function post-RT (Table 1). Ventricular torsion (Figure 7a) showed a non-significant decrease post-RT compared to the sham rats. Diastolic cardiac function was affected post-RT (Figure 7b), where the early-diastolic strain rate significantly decreased in all directions at 8 weeks and 10 weeks post-RT compared to the sham rats. Furthermore, the radial diastolic strain rate significantly reduced at 10 weeks post-RT compared to 8 weeks post-RT. As might be expected after high-dose radiation to the heart, myocardial mechanical dyssynchrony increased post-RT (Table 1 and Figure 7c), where radial TTP significantly increased at 8 weeks post-RT compared to sham rats, while both circumferential and radial TTP significantly increased at 10 weeks pot-RT compared to sham rats. Furthermore, circumferential TTP significantly increased at 10 weeks post-RT compared to 8 weeks post-RT. 

## 4. Discussion

In this study, we investigated the effect of localized heart irradiation on myocardial contractility patterns by studying different MRI-generated parameters that measure different aspects of systolic and diastolic functions, as well as mechanical dyssynchrony. The results demonstrated the sensitivity of the newly developed parameter, ContractiX, for early detection of subclinical cardiac dysfunction post-RT despite normal LVEF.

The results from this study illustrate the multifaceted effect of RT on myocardial contractility. Many reports, both clinically and pre-clinically, measure RT-induced cardiotoxicity utilizing LVEF as a measure of cardiac function [15,16,26,31,32,33,34]. Despite its value, LVEF mainly reflects global cardiac function, typically affected at a later time point post-RT. However, parameters of regional cardiac function can be measured for early detection of RT-induced cardiotoxicity. From this perspective, regional myocardial strain can be used to calculate the percentage of normally contracting myocardium (ContractiX), which serves as an early detector of the RT effect on heart function. The increase in LVEF post-RT could be attributed to undergoing ventricular remodeling and hypertrophy to maintain cardiac output in the face of RT injury.

Our results demonstrate the value of ContractiX as an early marker of subclinical cardiac dysfunction post-RT despite normal global function (Figure 6). While normal LVEF was maintained post-RT, ContractiX showed a continuous decrease in values from the sham-treated rats to 8 weeks post-RT rats and then to 10 weeks post-RT rats. ContractiX parameters calculated from circumferential strain, both circumferential and longitudinal strains, or all three strain components demonstrated significant reductions between 8 weeks and 10 weeks post-RT (Figure 4), which presents ContractiX as a sensitive marker of cardiac dysfunction progression post-RT, especially since all ContractiX parameters demonstrated greater and more significant reductions post-RT compared to strain reductions (Figure 5). When ContractiX was examined versus LVEF on a scatter plot, the results identified a nonlinear inverse relationship between the two parameters (Figure 6), where ContractiX spanned a large range of values between 25% and 100% among different rat groups despite normal LVEF (≥59%) in all rats. It is possible that ContractiX may hold promise for risk stratification of cancer patients receiving higher incidental heart radiation exposure as part of their cancer treatment, for example, in lung and esophageal cancer patients. Ultimately, this could lead to prompt initiation of cardioprotective therapy before global heart function is affected to avoid clinical cardiac dysfunction and subsequent heart failure.

There are three unique features of the introduced ContractiX parameter: (1) it can be calculated based on different strain components or combinations of these strain values; (2) it is agnostic to the strain acquisition sequence and analysis technique; and (3) the threshold values are determined utilizing the mean and standard deviations of strain measurements from normal subjects in the population being investigated for accurate differentiation between normal and pathological cases. The last feature is important as normal strain ranges could be different in different studied groups, species or even based on the strain acquisition and analysis techniques.

Our results also demonstrate the effect of RT on other parameters of heart mechanics. For example, ventricular torsion showed a small, non-significant decrease post-RT. While this parameter did not distinguish early changes post-RT well in this study, it may be useful to compare individuals in other situations, e.g., with different doses or time points post-RT. The study showed that RT affects not only systolic function but also diastolic function [25,33]. This was represented by the reduced early-diastolic strain rate, which was significant for all strain components at 8 weeks and 10 weeks post-RT. Diastolic cardiac dysfunction could be due to tissue stiffening secondary to changes in myocardial tissue composition post-RT, e.g., the presence of interstitial and perivascular fibrosis, myocardial vacuolization, and/or necrosis, as previously illustrated in preclinical models [15,26] and shown to be an important mechanism of radiation-induced heart dysfunction [35]. Our study also demonstrates that RT affects not only contractility magnitude but also contractility timing. The results show that while different myocardial segments contract at almost the same time point (time-to-peak strain) in the healthy heart, this mechanical synchrony is affected by radiation such that the variation of time-to-peak strain between different myocardial segments increases at 8 weeks and 10 weeks post-RT (Figure 7c). The induced mechanical dyssynchrony constitutes an additional factor contributing to the weaker contractility performance post-RT. It should be noted that while ventricular torsion, diastolic strain rate, and mechanical dyssynchrony showed altered measurements post-radiation, they were not as sensitive as the proposed ContratiX parameter. Specifically, all ContractiX parameters (Figure 4) showed statistically significant reduction not only between sham and RT rats but also between 8 weeks post-RT and 10 weeks post-RT, which was not the case for other parameters shown in Figure 7. For example, ventricular torsion (Figure 7a) showed very close values in different rat groups without any significant differences between the groups. Furthermore, while diastolic strain rate (Figure 7b) and mechanical dyssynchrony (Figure 7c) showed significant differences between the sham and RT rats, they showed limited capability for differentiating between the 8 week post-RT and 10 week post-RT. Therefore, the additive value of the proposed ContractiX parameter is its higher sensitivity for detecting slight changes in heart contractility during different time points in the study, which may not be feasible using other cardiac measures and allows for a prompt intervention to avoid advancement to heart failure.

Our study has some limitations. The first limitation is the small number of animals used in the study. The main purpose of this study was to introduce the new measure, ContractiX, and provide a proof-of-concept about its sensitivity for detecting subclinical cardiac dysfunction, which was demonstrated by the results despite the small number of animals. However, future studies on a larger cohort are warranted to confirm these results with sufficient statistical power.

Another limitation of our study is that we did not include another group of salt-resistant rats. The choice of salt-sensitive rats for this study was based on their cardiovascular profile, which is more vulnerable to cardiac injuries [16,26,36] (radiation in this study) compared to the more immune salt-resistant rats, which makes it appropriate for the investigation in this study about comparing different MRI measures of cardiac function. However, studying the effect of rat strain (comparing salt-sensitive to salt-resistant rats) on the performance of the developed ContractiX parameter is warranted in future research.

Another limitation of the current study is the unavailability of perfusion or late gadolinium enhancement (LGE) MRI data. However, the focus of this study was to characterize the cardiac function and myocardial contractility patterns shortly after RT and before global heart function is affected, where ischemia development and myocardial infarction are not expected to occur during the acute phase post-RT. Nevertheless, larger studies with longer follow-up times are warranted to confirm the results from this study and evaluate late RT effects on the heart. Although the focus of this study was on situations where the heart receives large radiation doses based on the tumor’s proximity to the heart (such as in lung and esophageal cancers), the presented imaging biomarkers may be valuable in other cancer types that incur incidental heart irradiation, e.g., in breast cancer or lymphoma, and they should be evaluated in these types of exposures in the future.

There is great potential for the translational value of this study, especially in cases of significant radiation to the heart when the tumor’s location is close to the heart [8,37]. The implementation of the developed technique in clinical studies would allow for earlier detection of subclinical cardiac dysfunction that may not be detected using conventional cardiac MRI measures. Such capability would be valuable for risk stratification, prognosis, monitoring, and management of cancer patients. Based on the demonstrated sensitivity of ContractiX, it can be used for detailed assessment of baseline cardiac function even before treatment starts. Based on the information obtained about ‘weak’ heart regions that show borderline contractility, treatment planning could be optimized to minimize the radiation dose delivered to these regions. Follow-up scans would also be valuable for identifying patients at risk in whom cardioprotective therapy could be promptly initiated to avoid progression into heart failure. Another advantage of the proposed technique is that it does not require using a gadolinium contrast agent, which is important from a safety perspective, especially in patients with compromised kidney function where it can cause nephrogenic systemic fibrosis (NSF) and because of gadolinium deposition in other organs, including the brain [38]. Furthermore, as the imaging biomarkers presented in this study provide information about regional heart function, they could be valuable in future studies to investigate the relative radiation sensitivity of different cardiac substructures or myocardial segments, which may ultimately lead to improved RT planning and better outcomes. Moreover, a variety of cancer-related pharmacologic treatments result in cardiomyopathy, which is frequently preceded by alterations in myocardial strain. Thus, the use of ContractiX could be tested after other cancer treatments for its utility in identifying early subclinical cardiac dysfunction and treatment management.

Finally, it should be noted that cardiac MRI provides a plethora of techniques for the comprehensive evaluation of heart health, including assessment of systolic function, diastolic function, hemodynamics, and tissue characterization [39]. Although different techniques reflect different aspects of the heart condition, ContractiX allows for early detection of subclinical cardiac dysfunction during ventricular remodeling when other parameters, e.g., ejection fraction, are still within the normal range. While a comprehensive evaluation of the heart function is always desirable, it may not be feasible in lung cancer patients who are at the advanced cancer stage and have limited capability of enduring a long comprehensive cardiac MRI exam that requires several breath-holds. In this case, an optimized short cardiac MRI exam that includes a few sequences for evaluating heart contractility and measuring ContractiX would be desirable and sufficient for risk stratification and prognosis in this patient population.

## 5. Conclusions

The results from this study characterize the effect of RT on myocardial contractility patterns and demonstrate the value of the newly defined ContractiX parameter as a sensitive early marker of subclinical cardiac dysfunction post-RT, that might be superior to other measures of cardiac function, such as LVEF and strain, if proved by further studies.

## Figures and Tables

**Table 1:** Myocardial cardiac function measurements in sham and irradiated. The strain-derived parameters, especially ContractiX, show reduced cardiac function post-RT.

	Sham	8-Weeks Post-RT	*p* (8 Weeks vs. Sham)	10-Weeks Post-RT	*p* (10 Weeks vs. Sham)	*p* (8 Weeks vs. 10 Weeks)
EF (%)	67 ± 6.9	78 ± 1.8	<0.01	77 ± 1.7	<0.01	0.37
EDV (mL)	0.29 ± 0.02	0.26 ± 0.03	0.05	0.27 ± 0.02	0.06	0.38
ESV (mL)	0.1 ± 0.02	0.06 ± 0.01	<0.01	0.06 ± 0.01	<0.01	0.30
SV (mL)	0.2 ± 0.02	0.2 ± 0.02	0.71	0.2 ± 0.01	0.42	0.52
Mass (g)	0.38 ± 0.04	0.49 ± 0.05	<0.01	0.56 ± 0.04	<0.01	0.04
Ecc (%)	14.1 ± 2.2	10.4 ± 0.5	<0.01	8.2 ± 0.4	<0.01	<0.01
Err (%)	23.3 ± 4.0	22.5 ± 3.6	0.71	17.8 ± 4.5	0.06	0.11
Ell (%)	15.6 ± 1.8	12.0 ± 0.7	<0.01	12.0 ± 1.9	<0.01	0.99
ContractiX-circ (%)	74 ± 20.9	44 ± 6.3	<0.01	21 ± 3.4	<0.01	<0.01
ContractiX-rad (%)	56 ± 14.4	50 ± 18.2	0.54	34 ± 16.9	0.04	0.09
ContractiX-long (%)	62 ± 15.9	40 ± 9.1	0.01	37 ± 13.9	0.02	0.62
Torsion (°/mm)	1.5 ± 0.5	1.4 ± 0.5	0.67	1.3 ± 0.4	0.43	0.86
SR-cc (1/s)	3.7 ± 0.26	2.2 ± 0.27	<0.01	1.8 ± 0.43	<0.01	0.17
SR-rr (1/s)	4.6 ± 0.63	2.9 ± 0.32	<0.01	2.5 ± 0.38	<0.01	0.04
SR-ll (1/s)	2.4 ± 0.61	1.8 ± 0.24	0.03	1.7 ± 0.41	0.03	0.73
TTP-cc (ms)	6.6 ± 2.6	15.4 ± 8.0	0.19	12.2 ± 4.4	0.04	0.40
TTP-rr (ms)	6.7 ± 2.5	13.6 ± 5.2	0.04	13.8 ± 4.3	0.02	0.96
TTP-ll (ms)	7.4 ± 3.5	11.8 ± 7.7	0.28	10.9 ± 2.6	0.08	0.81

Abbreviations: Ecc, Err, Ell: circumferential, radial, and longitudinal strains SR-cc, SR-rr, SR-ll: circumferential, radial, and longitudinal early-diastolic strain rates, respectively. TTP-cc, TTP-rr, TTP-ll: standard deviation of circumferential, radial, and longitudinal time-to-peak strain, respectively. Values represented as mean ± SD. *p*-values calculated vs. sham. Strain and strain rate measurements are represented in absolute value form (i.e., originally negative Ecc, Ell, and SR-rr measurements are represented as positive values).

## Data Availability

Study data is available upon request from the corresponding author.
